# Lifestyle Interventions in Patients in Active Surveillance for Prostate Cancer: A Systematic Review

**DOI:** 10.3390/jcm15093369

**Published:** 2026-04-28

**Authors:** Marco Campetella, Francesco Pio Bizzarri, Pierluigi Russo, Riccardo Bientinesi, Giovanni Battista Filomena, Maria Chiara Sighinolfi, Bernardo Rocco, Emilio Sacco

**Affiliations:** 1 Department of Urology, Ospedale Isola Tiberina-Gemelli Isola, 00168 Rome, Italy; 2 Department of Urology, Fondazione Policlinico Universitario Agostino Gemelli, IRCCS, 00168 Rome, Italy; 3 Department of Urology, Mater Olbia Hospital, 07026 Olbia, Italy; 4 Department of Medicine and Translational Surgery, Università Cattolica Del Sacro Cuore, 00168 Rome, Italy

**Keywords:** active surveillance, prostate cancer, lifestyle intervention, exercise, diet quality

## Abstract

**Background/Objectives**: Active surveillance (AS) has become the gold standard for managing men diagnosed with low-risk or favorable intermediate-risk prostate cancer. However, both patients and healthcare providers often face a lack of clear, evidence-based guidance regarding lifestyle choices during this period. This systematic review was designed to determine whether specific lifestyle modifications—including dietary changes, physical activity, weight control, and use of supplements—can tangibly impact oncologic outcomes or improve patient-reported quality of life during surveillance. **Methods**: The research followed PRISMA protocols, searching PubMed, Cochrane, and Scopus for studies published between 2000 and 2025. The team included diverse methodologies, from randomized controlled trials to qualitative interviews, specifically focusing on men on AS. To ensure high standards, two independent reviewers performed data extraction and quality assessments using CASP tools, and the review was formally registered with PROSPERO. **Results**: The review synthesized data from over 30 heterogeneous studies. The findings suggest that lifestyle interventions are safe and highly feasible. Physical exercise emerged as the most effective intervention, consistently improving cardiorespiratory fitness and reducing psychological burdens such as fatigue and “PSA anxiety.” While dietary changes and weight loss successfully improved metabolic health markers, they did not show a consistent ability to prevent biopsy upgrading or MRI progression. Similarly, supplements showed only minor, short-term effects on PSA kinetics without providing reproducible oncologic protection. **Conclusions**: For men undergoing active surveillance, lifestyle interventions may be considered as supportive measures, as they appear feasible and may improve physical fitness, metabolic health, and selected patient-reported outcomes. However, current evidence remains insufficient to demonstrate a consistent effect on biopsy upgrading, MRI progression, or long-term deferral of definitive treatment.

## 1. Introduction

Prostate cancer (PCa) is the most commonly diagnosed non-cutaneous malignancy in men and remains a major cause of cancer-related mortality worldwide [1]. Advances in prostate-specific antigen (PSA) testing, multiparametric magnetic resonance imaging (mpMRI), and targeted biopsy have increased detection of low-risk and selected favorable intermediate-risk disease. For most of these patients, active surveillance (AS) is now the preferred management strategy, allowing defer [1,2]. Compared with surgery or radiotherapy, AS avoids treatment-related morbidity—particularly urinary incontinence, erectile dysfunction, and gastrointestinal toxicity [3]. Nevertheless, longitudinal data indicate that up to 60% of men on AS ultimately undergo definitive treatment, most often due to pathological upgrading or increased tumor volume [3]. As the number of patients managed with AS has grown, interest has likewise increased in adjunctive, lifestyle-based approaches that might delay disease progression, extending the surveillance period without compromising cancer control. Translating AS from protocol to everyday life, however, exposes a substantial gray area: what, concretely, should a man do while he is being “watched” rather than treated? Current clinical guidelines largely refrain from issuing actionable, evidence-based lifestyle recommendations. There is no consensus on appropriate dietary patterns or restrictions; the intensity and frequency of physical activity; or best practices for weight control, sleep hygiene, alcohol intake, and supplement use during AS. As a result, counseling remains heterogeneous across clinicians and centers, leaving patients to navigate a fragmented information landscape of variable—and often uncertain—quality [1,4]. Patients commonly report a perceived inertia “doing nothing” that clashes with the urge to adhere to cancer prevention campaigns and to act after a cancer diagnosis. In the absence of standardized, trustworthy guidance, they may adopt unvalidated interventions, oscillate among contradictory advice, or discontinue health-promoting behaviors whose effects on oncologic outcomes remain uncertain. In parallel, the urologic literature highlights complementary and alternative medicine (CAM) as an area where clinicians should proactively guide patient choices, an issue that overlaps with lifestyle counseling during AS [5]. Psychological burden is another critical dimension. Living with an “untreated” cancer can generate cancer-specific anxiety and fear of progression, which ebb and flow with PSA fluctuations or the anticipation of re-biopsy. Importantly, distress has been cited as a reason some men exit AS in favor of definitive therapy despite stable disease, underscoring that tolerance of surveillance is not purely biological but also behavioral and emotional. Providing evidence-informed actions—rather than watchful inaction alone—may mitigate anxiety and improve persistence with AS [6]. This systematic review aims to comprehensively evaluate lifestyle modifications, such as dietary patterns and components, physical activity, weight management, and other behavioral factors, in men on AS for prostate cancer. Our objectives are to clarify the extent to which these behaviors influence disease trajectory (progression, treatment conversion, and oncologic safety) and the lived experience of surveillance (anxiety, quality of life, and adherence), and to identify evidence gaps and methodological priorities needed to inform future consensus statements and guideline development.

## 2. Materials and Methods

This systematic review was conducted in accordance with the Preferred Reporting Items for Systematic Reviews and Meta-Analyses (PRISMA) guidelines (Supplementary File S1) [7].

### 2.1. Search Methods

The research strategy involved collecting data from 3 databases: PubMed, Cochrane, and Scopus. We collected all articles within a time frame from 2000 to 2025. The search was conducted using Boolean operators and keywords as follows: we used “prostate cancer” OR “prostatic carcinoma” OR “prostatic neoplasma” AND “Life Style” OR “lifestyle” OR “diet” OR “nutrition” OR “physical activity” OR “exercise” OR “smoking” OR “alcohol” OR “obesity” OR “body weight” OR “BMI” OR “Mediterranean diet” OR “plant-based diet” AND “Active Surveillance” OR “active surveillance” OR “watchful waiting” AND “Risk Factors” OR “prevention” OR “progression” OR “prognosis” OR “survival” OR “recurrence”. This systematic review was prospectively registered in the PROSPERO international database of systematic reviews (registration number: CRD420251157972).

### 2.2. Inclusion Criteria

We included original qualitative and quantitative studies evaluating lifestyle-related interventions or exposures in men undergoing active surveillance for prostate cancer.

### 2.3. Exclusion Criteria

We excluded narrative reviews, systematic reviews, meta-analyses, conference abstracts, expert opinions, and case reports from the primary evidence synthesis. Protocol papers and secondary analyses of the same study program were considered separately for feasibility or contextual interpretation and were not treated as independent efficacy studies.

### 2.4. Screening Procedure

**Figure 1:**
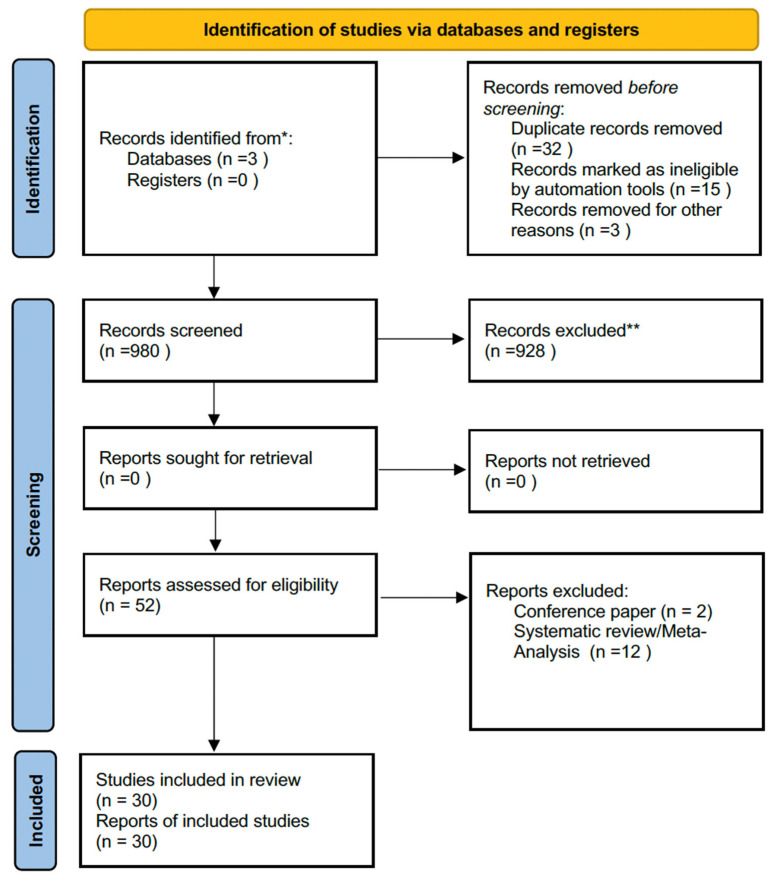
PRISMA 2020 flow diagram for new systematic reviews which included searches of databases and registers only. (** by two reviewers; * by one reviewer).

The screening process was performed independently by two reviewers, first assessing titles and abstracts, followed by full-text evaluation. To ensure comprehensive coverage, the reference lists of all eligible studies were manually searched for additional relevant publications. The study selection procedure is illustrated in Figure 1.

Methodological appraisal was performed independently by two reviewers using the design-appropriate CASP checklist. The appraisal informed interpretation of the evidence and is reported item by item in the Supplementary Materials.

From each full text, data were extracted on study design, disease grade, lifestyle factors, quality of life and oncological-related outcomes. Data extraction was carried out independently by two reviewers: one performed the primary extraction, and the second verified accuracy. Any discrepancies in data collection or quality ratings were resolved through discussion and consensus.

The study selection process including identification, screening, eligibility, and inclusion is summarized in the PRISMA 2020 flow diagram (Figure 1), which also details reasons for exclusion at each stage. Where the active surveillance subgroup size was not separately reported in the original article, this is explicitly indicated in Table 1 rather than treated as missing extraction (Table 1) [7]. Because the available literature included randomized trials, observational cohorts, feasibility studies, and qualitative reports, the evidence was synthesized in a stratified narrative manner according to study design and contribution to inference and because of substantial clinical and methodological heterogeneity across study designs, interventions, progression definitions, comparators, and reported outcomes, a quantitative meta-analysis was considered inappropriate and potentially misleading; therefore, a structured narrative synthesis was performed.

## 3. Results

The study selection process is summarized in Figure 1 (PRISMA 2020 flow diagram), while the characteristics of the included studies are reported in Table 1. The studies included in the primary synthesis were grouped according to intervention domain and narratively synthesized as follows: dietary patterns and dietary counseling, supplements and nutraceuticals, physical activity and structured exercise, and weight management/adiposity-related interventions.

### 3.1. Study Characteristics

Thirty studies were included in the primary synthesis, with substantial heterogeneity in design, intervention type, comparator definition, follow-up duration, and outcome reporting. The available evidence comprised randomized controlled trials, non-randomized interventional studies, observational cohorts, qualitative studies, and feasibility or protocol reports.

Interventions could be broadly grouped into:

- Dietary patterns and dietary counseling;
- Supplements and nutraceutical approaches;
- Physical activity and structured exercise interventions;
- Weight management, adiposity, and metabolic modulation.

Outcomes were also heterogeneous and included biopsy upgrading, pathologic reclassification, conversion to treatment, MRI-related or tissue-based biomarkers, PSA kinetics, inflammatory or metabolic markers, and patient-reported outcomes such as quality of life, anxiety, fatigue, and acceptability. Because linked publications from the same study program were present, these were considered together in the narrative synthesis rather than as independent efficacy datasets.

### 3.2. Dietary Patterns and Dietary Counseling

Evidence on dietary counseling in men on active surveillance was anchored by the MEAL program. The protocol and recruitment reports demonstrated the feasibility of a large multicenter dietary randomized trial in this setting, with successful enrolment across 91 sites and good baseline balance between arms [8,37,38]. In the primary efficacy analysis, the intervention increased vegetable intake but did not reduce short-term PSA- and biopsy-defined progression over 24 months compared with control. A subsequent secondary analysis showed no meaningful between-group differences in anxiety, urinary or sexual function, vitality, or overall health-related quality of life, largely in a population with high baseline HR-QOL and ceiling effects. Together, these reports suggest that intensive diet counseling is feasible and scalable, but did not demonstrate a clear disease-modifying effect within the follow-up available.

Observational studies evaluating overall diet quality yielded suggestive but inconsistent associations. In men enrolled on prospective active surveillance protocols, higher baseline HEI-2015 scores were associated with a borderline lower risk of Gleason grade progression, whereas no association was observed for dietary change over the first 6 months. Similarly, higher adherence to a Mediterranean dietary pattern was associated with a modest inverse trend for grade progression, while analyses from the Canary PASS cohort using HEI-2015, aMED, and DASH scores found no statistically significant associations with pathologic reclassification, despite weak inverse trends [17]. Coffee consumption also showed no clear association with progression, although the authors suggested that inter-individual biologic variability might be relevant. More recently, Su et al. extended this line of inquiry by examining diet quality and dietary inflammatory potential in relation to grade reclassification, further reinforcing interest in global dietary patterns rather than isolated nutrients [39].

Diet-related studies addressing feasibility and patient behavior highlighted an important implementation gap. Avery et al. showed that many men diagnosed with prostate cancer, particularly those managed conservatively, changed diet after diagnosis in order to “do something,” but wanted clearer and more credible professional guidance [40]. Ullevig et al. [41] reported the feasibility of dietary folic acid reduction in men on active surveillance, while early descriptive work by Daubenmier et al. suggested that lifestyle patterns and health-related quality of life are closely linked in this population [41]. In addition, Ornish et al. provided mechanistic pilot data suggesting that broader lifestyle change may influence telomerase-related biology in low-risk prostate cancer, although this evidence does not derive from a contemporary active surveillance efficacy framework [42]. Overall, diet appears acceptable to patients and biologically plausible, but consistent benefit on hard oncologic endpoints has not yet been shown.

### 3.3. Supplements and Nutraceutical Approaches

Supplement-based interventions were numerous but highly heterogeneous, and most relied on surrogate endpoints rather than standardized clinical progression outcomes. In the Pomi-T phase II trial, a polyphenol-rich whole food supplement was associated with a significantly smaller PSA rise over 6 months than placebo, and fewer men in the intervention arm left surveillance by study end [26]. In a later double-blind study, Thomas et al. also reported that adding a probiotic/prebiotic preparation to a phytochemical-rich supplement further slowed PSA progression and improved selected inflammatory markers, with possible favorable MRI trends in a subset of patients [27]. However, both studies primarily addressed biochemical or exploratory outcomes and cannot be interpreted as definitive evidence of reduced clinical progression.

Omega-3-related studies yielded a more complex picture. Chan et al. found that short-term supplementation with fish oil or lycopene did not significantly modify selected tissue gene-expression endpoints or PSA compared with placebo [43]. By contrast, observational biomarker studies by Moussa et al. and Moreel et al. suggested that higher intraprostatic EPA, rather than blood-based omega-3 measures, was associated with a lower probability of higher-grade disease or upgrading at confirmatory biopsy [21,28,44]. Aronson et al. further contributed two complementary signals: an interventional trial (CAPFISH-3) showing a reduction in intratumoral Ki-67 with a high omega-3/low omega-6 diet plus fish oil, but no significant difference in grade group, tumor length, Decipher score, or PSA after one year; and tissue biomarker data indicating that higher intraprostatic EPA may be associated with a lower risk of upgrading from GG1 to GG2. These findings support biologic plausibility, but not yet a consistent clinical benefit [20].

Evidence for vitamin D and other supplements was similarly mixed. Marshall et al. reported that vitamin D3 4000 IU/day for one year was safe and was associated with favorable repeat biopsy findings in some men, but the study was small, open-label, and partly supported by historical comparison only [18]. Campbell et al. observed that higher baseline vitamin D levels were associated with a greater likelihood of a downward PSA trend in men receiving a diet-plus-supplement regimen, whereas omega-3/omega-6 measures were not significantly correlated with PSA slope [30]. In contrast, Stratton et al. found no benefit of selenium on PSA velocity or PSA doubling time and raised the possibility of harm at high baseline selenium exposure [19]. Preclinical work by Bernichtein et al. suggested that vitamin D may counteract calcium-driven tumor-promoting pathways, but these data remain mechanistic and not directly translatable to clinical active surveillance endpoints [45,46]. Although most lifestyle interventions were feasible and generally safe, caution is warranted for unvalidated or restrictive approaches; selenium may be harmful at high baseline levels, metformin showed no overall progression benefit and possible subgroup harm in obese men, and extreme diets may be difficult to sustain long-term.

Finally, the phase III MAST trial showed no progression-free survival benefit with metformin and even suggested increased pathologic progression in obese men, underscoring that metabolic plausibility does not necessarily translate into oncologic benefit. Overall, supplement studies most often reported changes in PSA kinetics, tissue proliferation markers, or exploratory biomarkers, whereas reproducible effects on biopsy-confirmed progression or treatment conversion remain unproven [29].

### 3.4. Physical Activity and Structured Exercise

Exercise was the intervention domain with the most consistent signal for supportive benefit. Qualitative and feasibility studies showed that men on active surveillance are generally receptive to exercise-based support, although delivery preferences vary substantially. McIntosh et al. found that many patients overestimated their own activity levels, while expressing interest in individualized or group-based support to improve motivation, accountability, and confidence [47]. Protocol papers by Galvão et al. and Kang et al. also demonstrated that structured exercise trials in active surveillance are acceptable and operationally feasible, with planned endpoints spanning time to treatment, PSA-related measures, body composition, and patient-reported outcomes. Pre-EMpT similarly showed the feasibility of combining brisk walking with metabolic intervention strategies, although its mixed population design limits direct inference for pure AS cohorts [32,48,49,50].

Completed intervention studies consistently showed improvements in fitness and selected biologic or symptomatic outcomes. In the ERASE randomized trial, supervised HIIT improved cardiorespiratory fitness and yielded favorable changes in cardiometabolic biomarkers, with signal toward lower PSA-related measures and other tumor-associated surrogates. Van Blarigan et al. reported that a 12-week home-based walking intervention significantly improved VO_2_ peak and quality of life [33,51,52]. Hvid et al. [34] found that home-based endurance training improved physiologic function without adverse effects on PSA doubling time, while Moon et al. observed reduced inflammatory cytokines and improved quality of life in a pilot home-based exercise program [35]. Eriksen et al., in a combined whole-grain rye plus vigorous physical activity feasibility study, found improved peak VO2 but no significant PSA or metabolic changes, likely reflecting limited power. A small, randomized feasibility study comparing HIIT, resistance training, and usual care also showed acceptable adherence, with modest strength gains and selected biomarker changes, but recruitment remained challenging. Taken together, these interventional studies support exercise as feasible and beneficial for fitness, physiology, and selected patient-reported outcomes, but not yet conclusively for biopsy-based progression [12].

Observational data on physical activity and oncologic progression were mixed. Vandersluis et al. found that progressors tended to report less physical activity, but after multivariable adjustment, age was the only independent predictor [13]. Papadopoulos et al. found no significant association between post-diagnosis physical activity and time to discontinuation of surveillance in favor of curative treatment [14]. Similarly, some cohorts did not confirm an independent association between self-reported activity and reclassification. In contrast, Brassetti et al. [15] reported that higher PASE scores were independently associated with a lower risk of reclassification, while Guy et al. [24] found an inverse association between total or vigorous physical activity and reclassification, and Richman et al. reported that brisk or vigorous post-diagnosis activity was associated with reduced progression and prostate cancer-specific mortality [25]. Secondary evidence sources were directionally similar: Brassetti’s scoping review suggested an overall protective signal for physical activity, and Zhu et al.’s network meta-analysis of randomized trials supported supervised aerobic training for fitness and PSA-related surrogates, although these were not treated as independent primary studies in the present synthesis. Overall, exercise provides the clearest supportive care benefit, whereas evidence for a reproducible disease-modifying effect remains inconsistent.

### 3.5. Weight Management, Adiposity, and Metabolic Modulation

Weight-focused interventions suggested metabolic benefit, but only limited evidence for short-term oncologic impact. Kaiser et al. reported that a short ketogenic program in overweight or obese men on active surveillance achieved approximately 7.4% BMI reduction, with some favorable pathological observations at repeat biopsy, but no consistent change in PSA or inflammatory markers [9,53,54,55]. Wright et al. and the PALS program also support the feasibility of structured lifestyle modification in overweight or obese men, with modest weight loss and favorable insulin–IGF-related biomarker changes, but without convincing evidence of pathologic benefit over short follow-up [10]. Burton et al. further showed that lifestyle and anthropometric factors may influence repeat PSA levels during surveillance, emphasizing that metabolic exposures can affect PSA variability even in the absence of proven impact on tumor biology [31].

Observational studies of adiposity generally suggested that higher BMI is associated with less favorable pathology or more aggressive disease features, although not uniformly across cohorts. Ploussard et al. reported that higher BMI was associated with an increased risk of upstaged disease in men otherwise eligible for active surveillance. Bhindi et al. [56] and de Cobelli et al. [57] similarly linked obesity or higher BMI to adverse pathology, upgrading, or upstaging in low-risk disease. Jeong et al. found that obesity was independently associated with unfavorable disease and noted a possible relation to transition-zone tumor location, which may affect sampling accuracy and AS selection [58]. However, Merrick et al., in a transperineally staged cohort with long follow-up, did not find BMI to be an independent predictor of treatment conversion or quality-of-life trajectories, despite numerically higher intervention rates in heavier patients [59]. These findings suggest that excess adiposity is a plausible adverse host factor, but the strength and consistency of the association are limited by heterogeneous staging methods, progression definitions, and surveillance intensity across studies.

### 3.6. Overall Pattern of Findings

Across intervention domains, the most consistent benefits of lifestyle modification in active surveillance were observed for feasibility, fitness, selected metabolic parameters, and patient-reported outcomes such as fatigue, quality of life, and cancer-related worry. Exercise interventions showed the most reproducible supportive effects. Dietary interventions and weight loss approaches improved adherence targets or metabolic biomarkers more often than they altered biopsy-based progression. Supplement studies frequently reported changes in PSA kinetics, inflammatory markers, tissue fatty acids, or proliferation markers, but these effects were usually short-term and not consistently mirrored by harder clinical endpoints. Observational evidence also suggested that higher BMI may be associated with worse pathologic features, but results remained heterogeneous. Overall, the available literature supports lifestyle intervention as safe and potentially beneficial supportive care during active surveillance, while evidence for a consistent disease-modifying effect on prostate cancer progression remains limited and uncertain.

## 4. Discussion

### 4.1. Lack of Evidence-Based Lifestyle Recommendations

Contemporary AS protocols are increasingly standardized, typically incorporating serial PSA testing, digital rectal examination, mpMRI, and scheduled confirmatory or repeat biopsies [60,61,62,63]. Nevertheless, major guidelines (EAU, NCCN) stop short of prescriptive recommendations, both for detailed follow-up schedules and for diet, physical activity, or other behavioral measures. Although patients frequently seek such guidance, the available evidence remains limited and heterogeneous [8,16,18,23,64,65].

Notably, a narrative review on mpMRI underscored its utility not only for diagnosis but also to plan and monitor active surveillance, providing objective anchors when assessing lifestyle-driven changes in disease status [17,66].

This lack of firm direction reflects substantial methodological challenges: heterogeneous study designs, variable definitions of progression, and inconsistent interventions across studies. While biologic pathways associated with androgen modulation, systemic inflammation, and oxidative stress lend plausibility, robust clinical evidence is still sparse.

In a recent systematic review and meta-analysis, Matsukawa et al. synthesized 22 studies (six RCTs and 16 cohort studies) of non-surgical interventions in men on AS, spanning diet, physical activity, coffee consumption, and vitamin D supplementation. Dietary quality was evaluated using indices including HEI-2015, the Mediterranean diet score, and DASH [8,16,17,23,65,67]. Although several cohorts suggested potential protective associations against cancer progression, no consistent and reproducible benefit has been confirmed. Even the largest RCT, the MEAL trial, showed that counseling to increase vegetable intake did not meaningfully delay disease progression at 24 months versus controls [8].

This systematic review evaluates lifestyle interventions like dietary patterns and components, structured physical activity, weight management strategies, nutritional supplements, and related behavioral modifications, in men with low-risk or favorable intermediate-risk prostate cancer undergoing active surveillance. Across more than 50 heterogeneous studies, interventions were generally feasible and well tolerated, and most consistently improved cardiometabolic parameters and patient-reported outcomes (PROs), including aspects of anxiety, fear of progression, fatigue, and health-related quality of life. However, evidence for a reproducible effect on “hard” oncologic endpoints, like biopsy-confirmed upgrading, MRI progression, and durable deferral of definitive treatment, remains inconsistent and frequently underpowered, with follow-up commonly limited to 12–36 months. Consequently, lifestyle modification should be positioned as high-value supportive care and competing-risk optimization, with oncologic disease-modifying benefit plausible but not yet proven within contemporary AS pathways.

Biological plausibility is strong: lifestyle change may reduce systemic inflammation, improve insulin resistance and IGF axis signaling, modulate lipid mediators, alter immune surveillance, and influence tumor microenvironmental pathways. Yet, translating these mechanisms into clinically meaningful reductions in upgrading or treatment conversion likely requires adequate intervention “dose,” objective adherence verification, and longer follow-up than is typical of existing trials.

Overall, our synthesis converges with recent non-surgical intervention meta-analytic work highlighting heterogeneity of interventions and endpoints, with modest or inconsistent signals for delayed progression and more consistent benefits for fitness and quality of life [68]. Observational AS cohorts have reported mixed associations between higher Mediterranean diet adherence or higher overall diet quality and lower risk of upgrading or grade progression, with signals often borderline and susceptible to residual confounding and dietary measurement error. The prospective Mediterranean diet AS cohort literature (including the Gregg et al. AS cohort) supports a possible pattern-level signal rather than a single nutrient effect. [17]. In contrast, the large, pragmatic MEAL phase III trial demonstrated that a scalable, telephone-based intervention increasing vegetable intake did not reduce 24-month PSA-/biopsy-defined progression, even with objective adherence biomarkers (plasma carotenoids), underscoring the gap between short-term dietary change and measurable oncologic endpoints within two years.

Recent dietary-focused analyses have similarly examined Mediterranean-style adherence within AS cohorts; these studies collectively reinforce that any protective effect (if present) is likely modest, context-dependent (baseline risk, comorbid metabolic milieu), and sensitive to outcome definition and ascertainment strategy.

Trial evidence more consistently supports exercise for improving cardiorespiratory fitness and PROs during AS. The ERASE randomized trial program demonstrated improvements in VO_2_ peak and favorable changes in prostate cancer-specific anxiety and fear of progression (ref. [11] trial). The ASX trial likewise showed that a structured, home-based walking intervention can improve VO_2_ peak and reduce fear of recurrence and urinary obstruction/irritation, supporting feasibility and patient-centered delivery models [33]. These findings align directionally with supportive care meta-analytic evidence on exercise in prostate cancer populations [69,70], which emphasizes improvements in quality-of-life domains and functional outcomes, while effects on psychosocial subdomains may vary by intervention type, supervision intensity, and baseline distress.

Weight management is biologically compelling in AS because adiposity and insulin resistance may influence progression risk and complicate PSA interpretation. The PALS randomized trial demonstrated that clinically meaningful weight loss and improvements in insulin-related biomarkers are achievable in obese men on AS, yet short-term biopsy upgrading did not differ, consistent with the broader pattern that metabolic gains do not automatically translate into near-term oncologic differences.

Supplement trials often show short-term PSA or biomarker changes, but biopsy-confirmed benefits are inconsistent and tissue-level pathway modulation is variable. For example, short-term lycopene/fish oil supplementation did not clearly shift selected tissue gene-expression targets, whereas omega-3 status measured in prostate tissue may associate more strongly with aggressiveness than blood-based measures, highlighting biomarker selection as a key methodological determinant. The CAPFISH-3 trial is notable for using a tumor-biologic primary endpoint (Ki-67 change) and confirming exposure changes, while still showing limited differences in grade group or PSA, illustrating that biologic modulation may precede, and not necessarily guarantee, short-term clinical endpoint shifts.

Jeong et al.’s CaPSURE-based analyses are frequently cited to support the relevance of modifiable behaviors to PSA kinetics and AS discontinuation; the broader implication is that registry associations can generate hypotheses but remain vulnerable to confounding, outcome misclassification, and treatment-selection bias [71].

Finally, mechanistic syntheses emphasize that exercise and lifestyle exposures may influence cancer-relevant pathways, but translating these mechanistic insights to AS outcomes requires endpoint harmonization and longer follow-up [72].

Moreover, our final findings are also consistent with (1) the MEAL phase III trial (dietary counseling; null on 24-month progression), (2) ERASE (HIIT; fitness and psychosocial benefit), (3) ASX (home-based walking; fitness and fear-of-recurrence benefit), (4) PALS (weight loss; metabolic benefit without short-term upgrading difference), (5) Pomi-T (polyphenol-rich supplement; short-term PSA rise attenuation), and (6) the selenium AS trial (null effect on PSA velocity, potential harm in high baseline selenium), collectively illustrating why surrogate improvements often fail to translate into consistent clinical progression differences in short follow-up windows.

### 4.2. Explanations for Heterogeneity

High between-study variability and limited comparability likely reflect multiple, non-exclusive factors. First, “progression” is operationalized inconsistently across studies, ranging from PSA velocity or doubling time to MRI changes, biopsy upgrading, tumor volume measures, or conversion to active treatment; because these endpoints capture different biological processes and are variably shaped by patient anxiety and preferences, they cannot be treated as equivalent. Second, active surveillance pathways themselves differ across centers and eras, particularly with respect to confirmatory biopsy timing, the integration of mpMRI, the use of targeted versus systematic sampling, and whether pathology is centrally reviewed, all of which influence baseline risk classification and subsequent detection of change; in this context, occult higher-grade disease at enrolment can easily obscure any modest lifestyle-related effects. Third, interventions vary markedly in “dose” and implementation, including exercise intensity, supervision level, and delivery setting, as well as dietary targets and coaching intensity, while control participants frequently adopt lifestyle changes after enrolment, diluting between-group contrasts and biasing toward null results. Fourth, exposure assessment is often imprecise because many studies rely on self-reported diet and activity measures, whereas trials incorporating objective indicators, such as accelerometry, plasma carotenoids, or red blood cell/tissue fatty acid profiles, provide more credible verification and may partly explain discrepant findings. Fifth, observational estimates are vulnerable to confounding and effect modification, as baseline adiposity, metabolic syndrome, diabetes, concomitant medications (e.g., statins or metformin), and socioeconomic factors can influence both the capacity to adhere and the underlying risk of progression, yet covariate adjustment is variable and residual confounding is difficult to exclude. Finally, follow-up is frequently too short to capture differences in clinically meaningful endpoints in low-event-rate AS populations; if lifestyle exposures exert small cumulative effects, multi-year observation may be required before shifts in upgrading or treatment conversion become detectable.

The evidence supports integrating lifestyle counseling as a routine component of AS, primarily to improve overall health, reduce competing cardiometabolic risk, and improve tolerance of surveillance, while avoiding overstated claims of cancer control. Pragmatically, urologists should: (i) normalize AS-related uncertainty and proactively address surveillance-related anxiety; (ii) offer structured referral pathways (exercise oncology/physiotherapy, dietitian-led programs) rather than generic advice; (iii) document and review supplement use explicitly, given variability in quality and the potential for harm or false reassurance; and (iv) frame lifestyle change as adjunctive to protocolized monitoring, not a substitute for MRI/biopsy triggers.

Lifestyle modification can convert the perception of “doing nothing” into active coping and self-management, with measurable benefits in fitness, metabolic health, and multiple quality-of-life domains, and likely reductions in cancer-related worry for some men. Exercise programs with supervision or structured remote support appear particularly valuable for men with baseline distress or poor fitness, and may reduce PSA-related anticipatory anxiety and fear of progression [33]. Patients should be counseled that while dietary quality and exercise are beneficial and biologically plausible as tumor modulators, the current evidence base does not justify relying on lifestyle changes to avoid recommended surveillance steps or to delay treatment when progression criteria are met.

Conflicting findings across studies likely reflect differences in sample size, intervention intensity, follow-up duration, comparator quality, and endpoint selection. Positive signals were more often observed in small exploratory studies or in analyses focused on surrogate outcomes, whereas larger randomized or multicenter studies more frequently failed to demonstrate clear differences in biopsy-confirmed upgrading, MRI progression, or durable deferral of definitive treatment.

Although several lifestyle interventions were associated with changes in biologically plausible pathways, including inflammatory, metabolic, and insulin–IGF-related markers, these findings should be considered hypothesis-generating, as they have not yet been consistently linked to biopsy-confirmed upgrading, MRI progression, or treatment conversion in men on active surveillance.

### 4.3. Limitations

Primary study limitations include predominance of small, phase II and observational designs, short duration and follow-up, high attrition in some studies, inconsistent confounder adjustment, variable AS protocols, and frequent reliance on PSA-based surrogates without blinded adjudication. PSA kinetics were interpreted as surrogate outcomes and were considered separately from harder clinical endpoints, given the known biological variability of PSA and its imperfect correlation with disease progression during active surveillance. Diversity in race/ethnicity, socioeconomic strata, and BMI categories is often limited, restricting generalizability and effect modification analyses. Review-level limitations include unavoidable heterogeneity limiting quantitative pooling, risk of publication bias toward positive lifestyle findings, and outcome non-uniformity across the evolving mpMRI era. Exclusion of non-English/gray literature and reliance on aggregate published data restricts subgroup exploration and causal inference. Because of substantial heterogeneity in intervention type, comparator definition, follow-up duration, and outcome reporting, a formal meta-analysis was considered inappropriate and potentially misleading; therefore, a structured narrative synthesis was performed and a formal meta-analysis was not performed because the included studies were too heterogeneous in terms of interventions, populations, endpoints, and definitions of progression to support a reliable pooled estimate. Conflicting findings across studies likely reflect differences in sample size, intervention intensity, follow-up duration, comparator quality, and endpoint selection. In particular, positive signals were more often observed in small exploratory studies or in analyses focused on surrogate outcomes, such as PSA kinetics, inflammatory markers, or tissue biomarkers, whereas larger randomized or multicenter studies more frequently failed to show clear differences in biopsy-confirmed upgrading, MRI progression, or durable deferral of definitive treatment. Therefore, apparent inconsistencies should not be interpreted as evidence against lifestyle interventions per se, but rather as a consequence of substantial clinical and methodological heterogeneity across the available literature.

### 4.4. Implications for Future Research

Progress requires trials and cohorts designed to detect realistic effect sizes, minimize contamination, and harmonize outcomes across AS pathways. Multicenter pragmatic RCTs (≥3–5 years) embedded in AS clinics, with standardized mpMRI/biopsy schedules and central pathology review, should be prioritized to capture upgrading and treatment conversion reliably. Adaptive designs can personalize intervention intensity based on adherence or baseline metabolic risk; factorial designs can separate and test synergy between diet, exercise, and weight loss. Mechanistic substudies should incorporate paired tissue sampling with tracking (where feasible), metabolomics, inflammatory/insulin–IGF axis panels, and microbiome measures, explicitly linking objective adherence to biological change and then to clinical outcomes.

The minimum harmonized set for lifestyle trials in AS should include:

- Biopsy-confirmed upgrading (grade group change) using standardized definitions and central review;
- MRI progression using prespecified criteria and, ideally, central radiology review;
- Time to initiation of definitive treatment, with explicit categorization of reason (biologic progression vs. anxiety/preference);
- Longer-term metastasis-free survival via registry linkage when feasible.

Surrogate endpoints might include PSA kinetics reported in a standardized way (secondary, not primary), and tissue or validated circulating biomarkers aligned with the hypothesized mechanism (e.g., Ki-67 where tissue is sampled; insulin–IGF axis; inflammatory indices).

Overall, lifestyle interventions in men on active surveillance are best supported as structured supportive care that improves fitness, metabolic health, and multiple patient-reported outcomes, thereby strengthening long-term tolerance of surveillance and reducing competing-risk morbidity. Evidence that lifestyle change reliably delays biopsy upgrading, MRI progression, or treatment conversion remains inconsistent, largely because of heterogeneous endpoints, variable AS protocols, imperfect adherence assessment, and insufficient follow-up for low-event-rate populations. Urologic AS pathways should implement pragmatic, evidence-informed counseling now (emphasizing safety, adherence support, and supplement stewardship), while research advances toward longer multicenter trials using harmonized clinical endpoints, objective exposure verification, and integrated mechanistic substudies.

## 5. Conclusions

Across >50 heterogeneous studies in men with low-risk or favorable intermediate-risk prostate cancer on active surveillance, lifestyle interventions (dietary counseling, structured exercise, weight management and selected supplements) were feasible and generally safe. With moderate certainty, exercise programs improve cardiorespiratory fitness and some patient-reported outcomes (quality of life, fatigue, cancer-related worry), supporting lifestyle change as structured supportive care during surveillance. With low to very low certainty, evidence does not show a consistent, reproducible reduction in key oncologic outcomes such as biopsy upgrading, MRI progression or durable deferral of definitive treatment. Uncertainty reflects small samples, short follow-up, variable surveillance pathways, inconsistent progression definitions, control-group contamination and imprecise exposure measurement. Clinically, counseling should prioritize cardiometabolic risk reduction, psychological coping and safe behavior change, while discouraging reliance on supplements or lifestyle measures in place of protocolized MRI/biopsy monitoring. For men undergoing active surveillance, lifestyle interventions may be beneficial as supportive measures to improve physical well-being, selected patient-reported outcomes, and cardiometabolic health. However, current evidence remains insufficient to demonstrate a consistent effect on biopsy upgrading, MRI progression, or durable deferral of definitive treatment. Future multicenter pragmatic trials with harmonized endpoints, objective adherence verification and mechanistic substudies are clearly needed.

## Figures and Tables

**Table 1:** Summary of the 24 studies included in the systematic review. (EPA: eicosapentaenoic acid, CRF: Cardiorespiratory Fitness, NR: not reported, QoL quality of life, AS: active surveillance, HEI: healty eating index, aMED: Alternate Mediterranean Diet score; DASH: Dietary Approaches to Stop Hypertension, PA: phsycol activity; PSA: prostatic serum antigen, RBC: red blood cells, GG: Gleason Grade, BID: bis in die).

Study	Years	Design	N (AS)	Intervention/Exposure	Comparator	Duration	Primary Endpoints	Key Results/Notes
Parsons et al. [8] (MEAL Wol)	2020	Phase III RCT	Mixed population; AS-specific sample size not extractable	Intensive counseling to increase vegetable/carotenoid intake	Usual care	24 mo	Pathologic progression; conversion to treatment	No significant difference vs. control; QoL preserved (see 2022 analysis)
Kaiser et al. [9]	2024	Single-arm ketogenic weight loss program	Mixed population; AS-specific sample size not extractable	8-week ketogenic diet for overweight/obese men on AS	—	8 wk	BMI, PSA, inflammatory biomarkers; pathology at re-biopsy	~7.4% BMI reduction; several downgrading/remission; PSA/inflammation unchanged
Wright et al. [10] (PALS)	2024	Randomized lifestyle trial	Mixed population; AS-specific sample size not extractable	Combined diet + exercise program	Usual care	6 mo	Weight, insulin-related biomarkers; pathology	Significant weight loss and biomarker improvement; no pathologic change at 6 mo
Kang et al. [11] (ERASE)	2021	Phase 2 RCT	Mixed population; AS-specific sample size not extractable	12 weeks HIIT	Usual care	12 wk	CRF (VO2), PSA/PSA velocity, tumor growth indicators	Improved fitness; lower PSA/velocity; favorable tumor indicators; safe
Eriksen et al. [12]	2017	Randomized feasibility study	Mixed population; AS-specific sample size not extractable	Whole-grain rye-rich diet + vigorous PA	Usual habits	6 mo (+6 mo f/u)	Peak VO2; cardiometabolic indices; PSA	Increased peak VO2; no significant metabolic/PSA effects (likely underpowered)
Vandersluis et al. [13]	2016	Observational cohort	Mixed population; AS-specific sample size not extractable	Physical activity (self-reported)	—	NR	Progression/reclassification	No association
Papadopoulos et al. [14]	2019	Observational cohort	Mixed population; AS-specific sample size not extractable	Physical activity	—	NR	Progression/reclassification	No association
Brassetti et al. [15]	2021	Observational cohort (PASE scale)	Mixed population; AS-specific sample size not extractable	Physical activity level	—	NR	Progression/reclassification	Modest protective effect
Gregg et al. [16] (HEI-2015)	2019	Observational cohort	Mixed population; AS-specific sample size not extractable	Healthy Eating Index (diet quality)	—	NR	Gleason grade progression	Lower, non-significant risk with higher HEI
Schenk et al. [17] (Canary PASS)	2023	Observational cohort	Mixed population; AS-specific sample size not extractable	Diet scores: HEI-2015, aMED, DASH	—	NR	Pathologic reclassification	No significant associations; weak inverse trends
Gregg et al. [16]	2019	Observational cohort	Mixed population; AS-specific sample size not extractable	Coffee intake	—	NR	Progression	No clear association; genetics may matter
Marshall et al. [18]	2012	Open-label intervention	Mixed population; AS-specific sample size not extractable	Vitamin D3 4000 IU/day	—	12 mo	Biopsy cores; Gleason score	Fewer positive cores; stabilization/improvement in >50%; underpowered
Stratton et al. [19]	2010	Phase 2 RCT, double-blind	Mixed population; AS-specific sample size not extractable	Selenized yeast 200 or 800 µg/day	Placebo	≤5 y	PSA velocity; PSA doubling time	No benefit; 800 µg/day linked to higher PSA velocity (possible harm)
Aronson et al. [20]	2025	Randomized trial (tissue biomarker analysis)	Mixed population; AS-specific sample size not extractable	Higher tissue EPA levels	—	NR	Progression from GG1 to GG2	Higher EPA associated with lower risk of upgrading
Moreel et al. [21]	2014	Observational tissue biomarker study	Mixed population; AS-specific sample size not extractable	Prostatic EPA content; dietary/RBC omega-3s	—	NR	Progression (ISUP1)	Higher tissue EPA inversely associated; diet/RBC not predictive
Robles et al. [22]	NR	Feasibility RCT	Mixed population; AS-specific sample size not extractable	Brisk walking, metformin 500 mg, both	Usual care	6 mo	Randomization/adherence; AEs	Feasible; adherence ~47% (~60% pre-COVID); few AEs
Gregg et al. [23]	2023	Observational cohort	Mixed population; AS-specific sample size not extractable	Mediterranean diet score	—	NR	Gleason progression	Inverse association (HR 0.88 per unit)
David E Guy et al. [24]	2018	Observational cohorts	Mixed population; AS-specific sample size not extractable	Total/vigorous PA	—	NR	Reclassification	Inverse association; OR ~0.42
Richman et al. [25]	2012	Observational	Mixed population; AS-specific sample size not extractable	Brisk/vigorous PA post-diagnosis	—	NR	Progression and mortality	Reduced progression/mortality; 61% lower PCa-specific mortality with vigorous PA
Aronson et al. [20]	2024	Randomized/controlled (reported)	Mixed population; AS-specific sample size not extractable	High omega-3, low omega-6 diet + fish oil	Control diet	NR	Ki-67 proliferation index	15% decrease vs. 24% increase in control; *p* = 0.043
Thomas et al. (Pomi-T) [26]	2014	Phase II RCT	199	Polyphenol-rich whole food supplement	Placebo	6 mo	PSA progression	Significant reduction in PSA progression vs. placebo
Thomas et al. [27]	2026	Double-blind RCT	200	Phytochemical-rich foods + probiotics	Placebo	NR	PSA progression; Gut microbiome	Evaluation of the diet–microbiota axis on low-risk PCa progression
Moussa et al. [28]	2019	Observational survey	144	Omega-3 intake vs. tissue levels	—	NR	Prostatic tissue EPA/DHA levels	Correlation established between dietary intake and actual prostate tissue levels
Fleshner et al. [29] (MAST)	2025	RCT	407	Metformin 850 mg BID	Placebo	36 mo	Clinical/pathologic progression	Large-scale trial evaluating metformin’s metabolic effect on AS stability
Campbell et al. [30]	2021	Correlative analysis	NR	Vitamin D and omega-3 intake	—	NR	PSA kinetics	Joint impact of VitD and omega-3 on PSA doubling time/velocity
Burton et al. [31]	2012	Observational	NR	Lifestyle factors; anthropometrics	—	Longitudinal	Repeat PSA levels	Weight and diet factors influence PSA fluctuations during monitoring
Galvão et al. [32]	2018	Multicenter RCT	NR	Structured exercise program	Usual care	NR	Transition to active therapy	Evaluating if exercise can delay the need for radical treatment
Van Blarigan et al. [33] (AS-EX)	2024	RCT	52	Home-based walking intervention	Usual care	12 wk	CRF (VO2 peak); QoL	Significant improvement in cardiorespiratory fitness and quality of life
Hvid et al. [34]	2016	Intervention	70	Home-based endurance training	Usual care	24 mo	PSA doubling time; physiology	Improved physiological function; safe with no adverse effect on PSA
Moon et al. [35]	2022	Pilot study	20	Home-based exercise program	—	NR	Inflammatory cytokines; QoL	Reduction in systemic inflammation and improved psychological well-being
Ploussard et al. [36]	2012	Observational	NR	Body Mass Index (BMI)	—	NR	Upstaging/upgrading risk	Higher BMI significantly increases the risk of upstaged disease

## Data Availability

No new data were created or analyzed in this study.

## Supplementary Materials

The following supporting information can be downloaded at: https://www.mdpi.com/article/10.3390/jcm15093369/s1, File S1: PRISMA Checklist.

## Abbreviations

The following abbreviations are used in this manuscript:

PCa	Prostate cancer
PSA	Prostate serum antigen
AS	Active surveillance
Mp MRI	Multiparametric magnetic resonance
CAM	Complementary and alternative medicine
PSAdt	PSA doubling time
QoL	Quality of life
GS	Gleason score
GG	Grade group
PIN	Prostatic intraepithelial neoplasia
RP	Radical prostatectomy
RCT	Randomized control trial
EPA	Eicosapentaenoic acid
CRF	Cardiorespiratory Fitness
